# The Effect of Intense Exercise on Equine Serum Proteoglycan-4/Lubricin

**DOI:** 10.3389/fvets.2020.599287

**Published:** 2020-12-16

**Authors:** Austyn Matheson, Suresh C. Regmi, Gregory D. Jay, Tannin A. Schmidt, W. Michael Scott

**Affiliations:** ^1^Biomedical Engineering Graduate Program, University of Calgary, Calgary, AB, Canada; ^2^Department of Emergency Medicine, Warren Alpert Medical School & School of Engineering, Brown University, Providence, RI, United States; ^3^Biomedical Engineering Department, University of Connecticut Health Center, Farmington, CT, United States; ^4^Department of Veterinary Clinical and Diagnostic Sciences, Faculty of Veterinary Medicine, University of Calgary, Calgary, AB, Canada

**Keywords:** exercise, proteoglycan-4, lubricin, equine, serum, racehorses

## Abstract

**Objective:** Local biological and biomechanical-stimuli modulate proteoglycan-4 secretion within synovial joints. For the horse, changes to proteoglycan-4 concentration and function are notable in acute joint injury and osteoarthritis. Proteoglycan-4 (also known as Lubricin) is present in the blood, however the effect of exercise on equine serum levels is unknown. The overall objective of this study was, therefore, to investigate the effect of intense exercise on serum proteoglycan-4 in thoroughbred horses.

**Methods:** Samples of blood were taken from thoroughbreds (*n* = 12) during a chuckwagon racing event (Alberta, Canada). The chuckwagon race is a sprint racing event where teams of horses pull a combined 1,325 lbs (601 kg) of wagon and driver around a 5/8th mile (1 km) of dirt track, racing at full gallop to the finish. Blood samples were collected 30-min before the race start, and several timepoints post-race: 5-min, 90-min, 3-h, 12-h, and 23-h. Proteoglycan-4 concentrations in serum were quantified by enzyme-linked-immunosorbent-assay using recombinant-human proteoglycan-4 standards and anti-proteoglycan-4 mAb 9G3. The molecular weight of immunoreactive proteoglycan-4 in serum was assessed by western blot.

**Results:** Proteoglyan-4 in serum demonstrated the expected high MW immunoreactivity to mAb 9G3, consistent with that of full length PRG4. Serum proteoglycan-4 decreased five-minutes post-race from baseline concentration (0.815 ± 0.175 to 0.466 ± 0.090 μg/mL, μ ± SEM, *p* < 0.01).

**Conclusions:** The concentration of serum proteoglycan-4 in horses decreased significantly five min post-exercise. A potential explanation for this finding could be increased proteoglycan-4 clearance from the circulation. Further investigations could extend to complete the detailed characterization of proteoglycan-4 structure and its potential function within the blood as it relates to joint health and exercise.

## Introduction

Regular exercise is essential to maintain mobility (1) and is commonly prescribed in moderation as a treatment for joint disease (2). Physical activity preserves cartilage volume and thickness (3) and can slow degeneration observed in acute joint injury (4), aging or articular disease (2). However, there is a lack of consensus on the optimal exercise program, duration, and intensity for health benefit. For the horse, an athlete by occupation, serological markers of skeletal tissue metabolism secreted in response to exercise have been studied with regards to bone and cartilage health.

Exercise-dependent skeletal metabolism markers within serum can be indicative of osteochondral tissue growth and degradation in the horse. These markers may be significantly affected by training, age, and joint health. For example, serum concentrations of the bone-metabolism biomarker osteocalcin, a protein which is produced by bone-forming osteoblasts, depend on horse age (5) and exercise training program duration (6, 7). Cross-linked telopeptide fragments for collagen-1 (CTX1), an indicator of bone degradation, are increased in serum after treadmill-training (8) and elevated in serum from stall-housed and treadmill-trained horses with and without early osteoarthritis (OA) (9). Cartilage oligomeric protein (COMP), a cartilage-growth indicator, decreases transiently within serum post-training (10). Reduced COMP in synovial fluid and urine from horses with a carpal bone fracture is also strongly correlated with serum levels (11). Levels of cross-linked telopeptide fragments collagen-2 (CTXII), which are indicative of cartilage degradation, are increased in serum and synovial fluid at various time points in an equine racing career (12). Lastly, keratan sulfate, an individual proteoglycan linked to aggrecan and a positive indicator for cartilage health, was reported to increase in foals and ponies in exercise (7, 13, 14). While these studies provide examples of osteochondral serum biomarkers that can be influenced by exercise, no studies to date have examined the effect of equine exercise on serum levels of proteoglycan-4 (PRG4), a protein that serves critical roles in cartilage lubrication and joint health.

PRG4, also commonly known as Lubricin, is a mucin-like glycoprotein with gene products located throughout the body as superficial zone protein (SZP), megakaryocyte-stimulating factor (MSF), and hemangiopoietin (HAPO) (15). In the articular joint, PRG4 is produced by chondrocytes in the superficial cartilage layer and synoviocytes of the synovium. PRG4 is a friction-reducing boundary-lubricant that minimizes cartilage wear and damage between articular interfaces (16). Both biological and biomechanical stimulation modulate PRG4 expression and abundance in the joint. Biologically, pro-inflammatory cytokines interleukin-1 (IL-1) and tumor necrosis factor-alpha (TNF-α) reduce PRG4 secretion (17), whereas transforming-growth-factor-beta (TGF-β) increases PRG4 expression (17, 18). Biomechanical loading can be chondroprotective as *in vitro* dynamic shear loads are PRG4-enhancing (19–21). However, static compression of cartilage explants did not alter PRG4 secretion in culture (21), and high levels of static loading decreased cartilage explant secretion (22). The effects of biomechanical loading and exercise on circulating levels of human PRG4 may have a dependence on the activity and the overall condition of the joint; serum PRG4 increased in runners and cyclists (23) and decreased in patients with OA half an hour after a treadmill walking exercise (with increases to serum COMP, matrixmetalloproteinasis-3, cleavage type II collagenases, and C-propetide II) (24). For the horse, joint injury and disease may result in increased PRG4 in synovial fluid (25–29), altered PRG4 gene expression (increased in synovium, decreased in cartilage) (27), with mixed effects on synovial fluid composition (PRG4 concentration), all which may contribute to inefficient lubrication (28, 29). Changes to PRG4 concentration and function in synovial joints are notable and critical in injury and disease; however, the influence of exercise on circulating levels of PRG4 in equine serum is unknown.

The overall objective of this study was to characterize serum PRG4 from thoroughbred horses after intense exercise. Dynamic biomechanical stimulation of articular tissues, both *in vitro* and *in vivo*, increases synthesis and synovial PRG4 concentrations, respectively. We hypothesized that serum PRG4 concentrations would increase following intense exercise.

## Materials and Methods

Approval for collection and use of equine samples was obtained from the University of Calgary Veterinary Sciences Animal Care Committee (Protocol AC16-0210**)**. Animals were physically examined for evident signs of lameness. All animals participating in the racing event were of good health. Hydration levels were not strictly monitored. With owners' consent, blood samples were collected by a board-certified equine surgeon (WMS) during and after a chuckwagon racing event. Subjects were a homogeneous group of thoroughbred geldings ranging from 4 to 13 years of age, Table 1. Concentrations of PRG4 in equine serum were quantified using full-length recombinant human PRG4 (rhPRG4) standards. rhPRG4 was obtained from Lubris BioPharma, LLC (Framingham, MA, USA) (30).

**Table 1:** A summary of the racehorses included in this study.

**Samples**	**Clinical Pre-Evaluation**	**Ages**	**Sex**	**Breed**
(*n* = 12)	Normal	4–13	Gelding	Thoroughbred

### Sample Collection

The chuckwagon race is an equine sprint event where teams of four horses pull a combined 1,325 lbs (601 kg) of wagon and driver around a 5/8th mile (1 km) of dirt track while racing at full gallop for ~1 min 20 s. Samples were collected 30-min before the race start and several timepoints post-race: 5-min, 90-min, 3-h, 12-h, and 23-h. Blood was collected from the jugular vein by venipuncture (vacutainer serum glass red top; Becton Dickinson, Franklin Lake, NJ, USA). Blood samples were kept at room temperature (RT) for 0.5–1 h to allow clot formation. Separated serum was transferred to a fresh tube and kept at 4°C for transportation and processing (≤1 h). Samples were then centrifuged (3000 x G, 30 min, 4°C) and the pellet discarded. Samples were separated into multiple aliquots and stored at -80°C.

### PRG4 Western Blot

**Figure 1:**
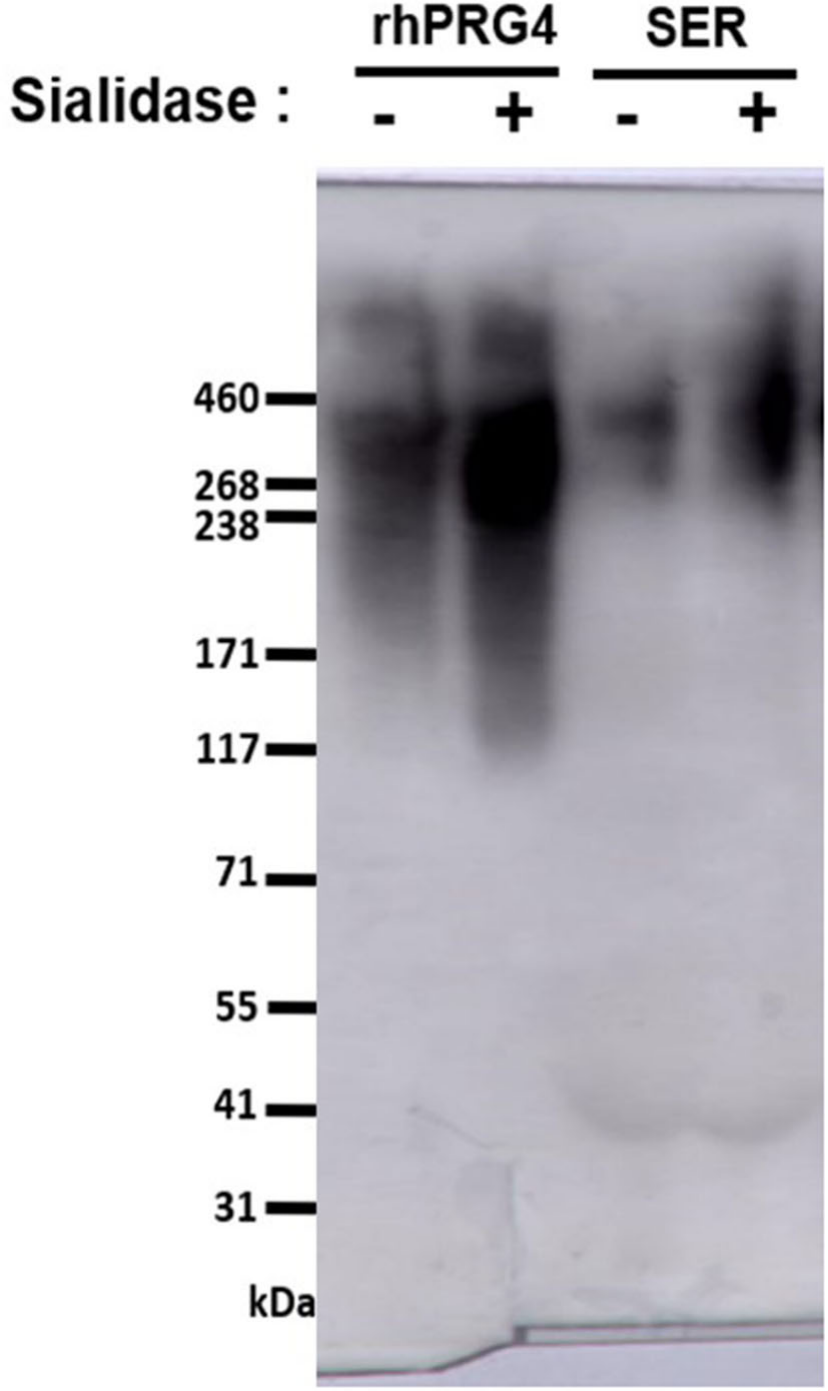
Western blot to assess serum PRG4 immunoreactivity to mAb 9G3. Samples were treated with dithiothreitol, and either with or without sialidase treatment.

Equine serum full-length PRG4 reactivity to the monoclonal antibody (mAb) 9G3 (Millipore Sigma, Etobicoke, Canada) was confirmed by western blotting using 3–8% SDS-PAGE, as described previously (25, 31). The apparent molecular weight of serum PRG4 is comparable to that of rhPRG4 of ~460 kDa (31, 32). All serum samples (and the rhPRG4 control) were treated with or without Sialidase-A (33) (ProZyme, Hayward, CA, USA), then electrophoresed under reducing conditions (5 mM dithiothreitol (DTT), NuPAGE™ Sample Reducing Agent, Thermo Fisher Scientific, Mississauga, ON, CA) (Figure 1).

### PRG4 Competitive Enzyme-Linked-Immunosorbent-Assay (ELISA)

Serum PRG4 concentrations were determined by competitive ELISA, as described previously (25). Briefly, the ELISA included the use of mAb-9G3, rhPRG4 standards, and a pre-treatment of serum and rhPRG4 standard with Sialidase-A (33) (ProZyme) at 37°C, overnight. The plate (96-well EIA/RIA Corning plate, Millipore Sigma) was pre-coated with 100 μL untreated rhPRG4 (10 μg/mL) overnight at 4°C. Contents of wells were rinsed 3x in PBS and blocked with 5% BSA TBST (2 h, RT). Separately, dilutions of sialidase-treated serum (dilutions of 1:1.5 and 1:2) and rhPRG4 standard (0–4 μg/mL) were incubated with 10 mM dithiothreitol (30 min, 56°C). These samples were incubated with biotin-conjugated mAb-9G3 (b-mAb9G3, 0.2 μg/mL) at a 1:1 ratio (1 h, RT). The well-contents (block solution) were discarded, and 100 μL of standard+b-mAb9G3 or sample+b-mAb9G3 was added to wells (1 h, RT). The plate then was rinsed 3x in TBST before adding 100 μL horseradish-peroxidase-conjugated-streptavidin (Millipore Sigma) in PBS (1:1000; 1 h, RT). The plate was rinsed 3x in PBS, developed (8 min) with 100 μL tetramethylbenzidine (Millipore Sigma), stopped with 25 μL of 1N H_2_SO_4_, and then read on a SpectraMax i3 reader (Molecular Devices).

### Statistical Analysis

Data were analyzed using a linear mixed-effects model to account for repeated sampling. Subjects served as their own controls. The fixed effect in the model was time of collection, with subjects as random effects to account for the non-independence of observations. Serum PRG4 concentrations were right skewed and so values were log-transformed for better model fitting. The model assumptions were checked by examining the residual plot for normality, and the constant variances plot for homoscedasticity. All summary statistics and values represent non-transformed data as mean ± SEM. In the case of significant fixed effect terms occurred, a *post hoc* analysis was performed using Dunnett's test to compare all post-baseline timepoints to baseline values. *p* < 0.05 was considered statistically significance. Statistical analysis was completed using R (R version 3.5.2, Platform: i386-w64-mingw32/i386, 32-bit) and R Packages including nlme, emmeans.

## Results

**Figure 2:**
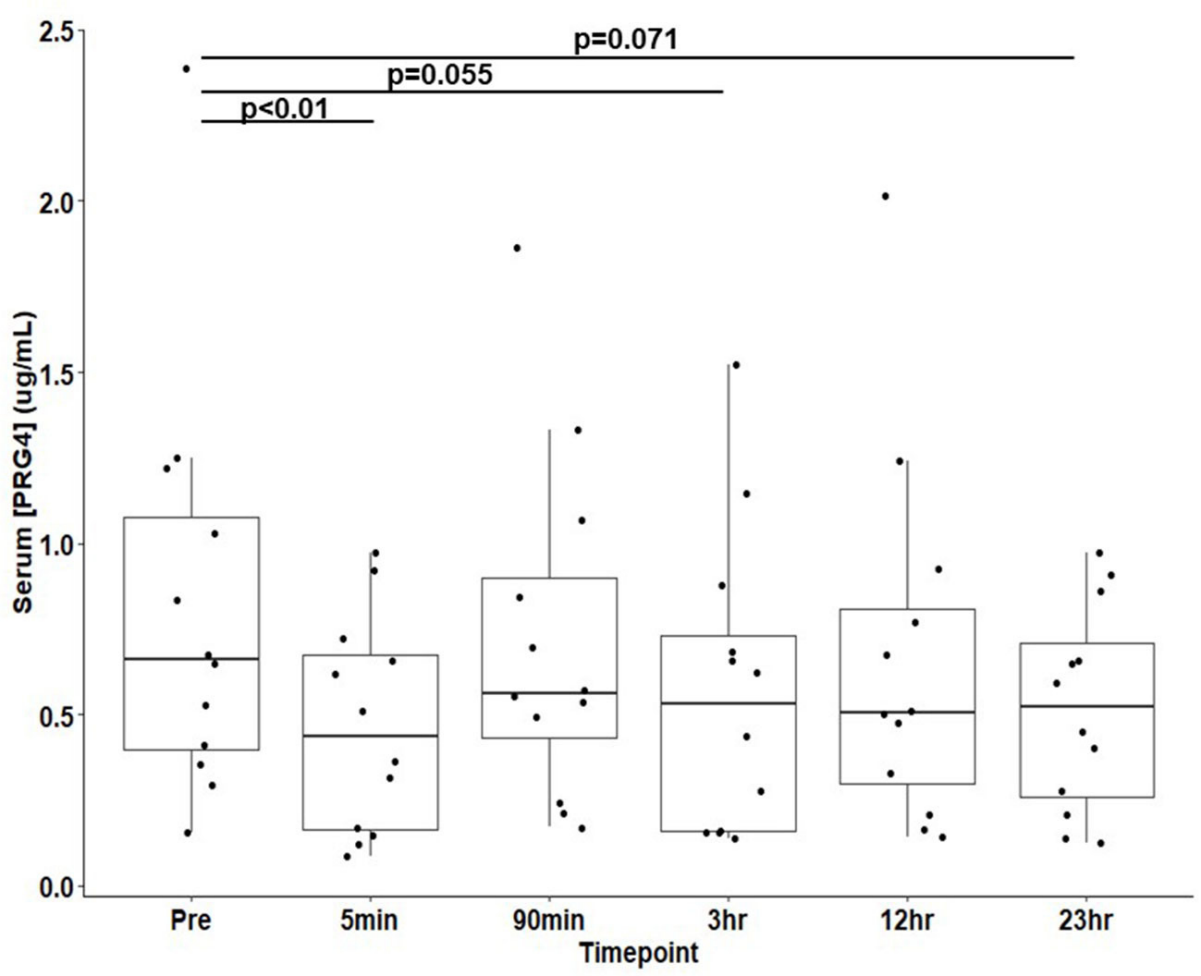
Serum PRG4 from thoroughbred horses (*n* = 12) at 30-min pre-race and five timepoints post-race. Data is presented as μ ± SEM.

PRG4 in serum demonstrated the expected high MW immunoreactivity to mAb 9G3, consistent with that of full-length PRG4 (Figure 1). In Figure 1, a broad mAb-9G3 immunoreactive band was observed with an apparent molecular weight of ~460 kDa. This molecular weight is consistent with that observed previously for PRG4 (31, 34). Also, the broad band observed is consistent with a previous study on synovial fluid PRG4 that employed a similar mAb to mAb-9G3 [mAb 5C11 (35)] that recognizes a combined sugar-peptide epitope (36). This observation was attributed to the electrophoretic behavior of a highly glycosylated molecule like rhPRG4 and the associated broad distribution of glycosylation-dependent epitopes (37, 38). The apparent molecular weight of serum PRG4 is comparable to that of rhPRG4 of ~460 kDa (31, 32). Treatment with sialidase appeared to increase the immunoreactivity of PRG4. Serum PRG4 concentrations decreased at five-minutes post-race compared to baseline (0.466 ± 0.090 μg/mL vs. 0.815 ± 0.175 μg/mL, *p* < 0.01, Figure 2). At later timepoints, changes in serum PRG4 were non-significant compared to the baseline concentration including sampling taken at 3-h (0.570 ± 0.128 μg/mL, *p* = 0.055) and 23-h (0.520 ± 0.087 μg/mL, *p* = 0.071).

## Discussion

The objective of this study was to investigate the effect of intense exercise on serum PRG4 concentration in thoroughbred horses. Serum PRG4 decreased five minutes post-exercise in all horses participating in this study. Overall, intense exercise resulted in a reduction of equine serum PRG4 concentration.

PRG4 concentration, structure, and synthesis are well-characterized in synovial joint tissues; however, little is known with regards to PRG4 origin and function in the blood. Limited studies are published on serum PRG4 concentrations within human subjects, and several examine its potential utility as a disease biomarker, particularly for joint disease. Superior biomarkers would be capable of detecting sensitive changes at the timepoints of joint disease where treatments are the most critical. In equine synovial joints, PRG4 increases with injury (26, 29) and disease (27) with potentially unrelated effects to serum (25). In human patients, serum PRG4 was unchanged in cases of rheumatoid arthritis (35) (0.53 to 1.41 μg/mL, measured by ELISA) and advanced joint disease (39) (0.25 to 1 μg/mL, measured by commercial ELISA kit), whereas plasma PRG4 was associated with joint space narrowing, after adjusting for age and sex (40). In contrast, for patients requiring total joint arthroplasty (TJA) serum PRG4 decreased alongside elevated serum cytokines IL-1β and TNF-α (39, 41). Collectively these findings suggest measurable changes to serum PRG4 concentration may lack specificity to joint disease.

In horses, strenuous exercise can be associated with increased inflammatory serum gene expression of IL-1 (2-h post-exercise) and TNF-α (6-h post-exercise) (42). In the present study, equine serum PRG4 decreased (Figure 2) immediately post-race. The immediate decline in serum PRG4 levels may relate to an apparent clearance or transfer to other tissues, possibly to the liver (43). However, apparent decreases in equine serum PRG4 samples (Figure 2) observed at 3 h and 23-h post-race could be associated with elevated serum cytokines after exercise downregulating PRG4 synthesis from the as yet to be determined source of serum PRG4. Future work, beyond the scope of this study, would be required to directly test hypothesis. Additionally, examination of serum PRG4 levels throughout the day of unexercised horses would be of interest for future study. Serum PRG4 levels are likely modulated by a combination of biological stimuli and cellular responses at the origin of production and secretion, in parallel to the effects of circulating factors and cytokines.

Local mechanical stimuli and moderate exercise affect synovial PRG4 synthesis. Given the role of PRG4 as a boundary lubricant and chondroprotective agent within the joint (16), exercise-associated effects on PRG4 cartilage metabolism could be informative to training regimes for equine athletes. In small rodents, *in vivo* synovial PRG4 increased with exercise intensity (20) and knee joint loading (44) by active cell responses, as opposed to mechanically-induced release. In human athletes participating in aerobic exercise running or cycling time trials of 30-min duration, a significant response to exercise was observed. Serum PRG4 increased immediately post-exercise (runners: from 0.104 to +39.4%, cyclists: from 0.118 to +56.9%, measured by commercial ELISA kit) (23). Conversely, for human subjects with medial knee osteoarthritis, serum PRG4 decreased by 43% after treadmill walking (both pre and post-operative intervention) (24). The present study has shown that equine serum PRG4 levels decrease acutely after intense exercise in agreement with Mündermann et al. (24), yet contradicts increases in serum PRG4 reported by Roberts et al. (23). These inconsistencies may be the result of joint condition differences, differences in exercise intensity and duration, or differences in sampling and analysis.

There are numerous technical challenges associated with characterizing serum proteins, including interference by serum constituents, including albumin, haptoglobin, transferrin, and lipoproteins (produced by hepatocytes) and immunoglobulins (IgGs, immune system) all found at very high concentrations (g/mL) in the blood. Previous works have characterized serum PRG4 by several formats, including commercial ELISA kit (23, 39, 45), ELISA plate format (35), and western blot (33). The commercially available PRG4 kit employed by previous studies utilizes a PRG4 antibody, of which the epitope is not provided by the manufacturer, in addition to variable serum PRG4 levels reported. In the present work, a modified ELISA plate format was used to detect full-length PRG4 (specific reactivity to mAb 9G3, visualized by western blot), with the serum being processed for optimal PRG4 detection. Serum PRG4 is extensively sialylated (46), and so samples were pre-treated with sialidase to increase reactivity to mAb 9G3 (33, 35) (Figure 1), then treated with DTT (10 mM) to inactivate serum IgMs (47), and finally heat-treated (48) (56°C, 30 min) to inactivate serum complement. Given the technical challenges mentioned above, as well as the variability in findings reported in the various studies, the techniques utilized for characterizing serum PRG4 should be considered when comparing values between studies, against species examined (varying baseline characteristics), as well as the antibody employed (varying affinity).

The mucin domain of PRG4 is extensively saturated with *O*-linked glycans that are partially capped with sialic acid. Detection antibodies with high affinity to the PRG4 mucin-domain, mAb 9G3 (35), S6.79 (33), and mAb 5C11 (36), have been shown to have some affinity dependency for the mucin domain post-translational modifications. The glycosylation profile of synovial PRG4 can vary with equine disease (49), which may affect the lubricating-ability of synovial fluid (47). The affinity of antibodies to PRG4 epitope can change with sialic acid modifications; for example, S6.79 reactivity to PRG4 epitope was shown to be highly masked by sialic acid in human samples including pericardium, splenic capsule, and trabeculae, plasma, serum, eye sleep extract, and liver, compared to synovial joint tissues (33). The reactivity of S6.79 to serum PRG4 increased by 3.9- to 14-fold after sialidase treatment (sialic acid cap removal). Interestingly, proteoglycans without sialic acid caps, asialoglycoproteins, are more readily cleared from the blood by lectin receptors in the liver (50). Given plasma PRG4 is modified extensively with O-linked sialylated glycans (46), additional sialic acid modifications present on serum PRG4 is one potential mechanism that may prevent clearance of PRG4 from serum to the liver. The mRNA expression of PRG4 in liver tissue is high, and particularly so in hepatocytes (46), which may indicate the liver as the origin of serum PRG4. Additional work could clarify the origin of PRG4, and distinct post-translational modifications, such as sialic acid caps, could aid in determining the location of synthesis or the function of PRG4 in the blood.

The horses used for chuckwagon events are a population of Thoroughbred geldings of similar age management and occupational history. The study group homogeneity in the present study is a significant strength in reducing possible confounding variables as the animals are from a group with high genetic similarity, athletic capabilities, and training, and in this case, ownership (with the same driver used for the entirety of the summer racing season). However, a resulting limitation in the participant selection is that these findings may not extend to other horse sex, breed, or age groups. The exercise challenge was also consistent between horses, with the animals harnessed together and running at the same speed, intensity, and duration. Before the racing event, a physical and standard lameness examination was performed on all horses in the study to confirm that they were sound and healthy. Still, individual differences in their overall state of health, injury, and medical history exist. Additional characterization of these differences, along with other variables such as the exact age, weight, and overall hydration, could address aspects of the natural variability of serum PRG4 measured in this study. The origin of serum PRG4 is unknown and is possibly unrelated to skeletal PRG4. Finally, according to the preliminary/pilot work, with an effect size of 0.445, to reach a power of 0.80 a sample size of 42 would have been required. We acknowledge that our study did not reach the desired power. However, with the existing limitations of our current study, we recruited the highest number of subjects possible.

Specific exercise regimes and biomechanical stimulation to articular tissues have been reported to increase synovial fluid PRG4. Therefore, the objective of this work was to examine the effect of exercise on equine serum PRG4, with the hypothesis that concentrations would increase in response. Significant decreases in equine serum PRG4 were observed following intense exercise, which did not support the study hypothesis. The noted decline in equine PRG4 may be due to the clearance or transfer of PRG4 to other tissues of the body. Future investigations could complete a detailed characterization of PRG4 origin, structure, and its potential function within the blood.

## Data Availability Statement

The raw data supporting the conclusions of this article will be made available by the authors, without undue reservation.

## Ethics Statement

The animal study was reviewed and approved by University of Calgary Veterinary Sciences Animal Care Committee. Written informed consent was obtained from the owners for the participation of their animals in this study.

## Author Contributions

AM: conceptualization, data collection, writing-original draft. SR: data collection. GJ: writing-reviewing and editing. TS: conceptualization, writing-reviewing and editing. WS: conceptualization, data collection, writing-reviewing and editing. All authors contributed to the article and approved the submitted version.

## Conflict of Interest

TS and GJ own equity in Lμbris BioPharma and have licensed patents related to the use of rhPRG4. TS also consults for Lμbris BioPharma. The remaining authors declare that the research was conducted in the absence of any commercial or financial relationships that could be construed as a potential conflict of interest.
